# Decoding the cognitive states of attention and distraction in a real-life setting using EEG

**DOI:** 10.1038/s41598-022-24417-w

**Published:** 2022-11-30

**Authors:** Pallavi Kaushik, Amir Moye, Marieke van Vugt, Partha Pratim Roy

**Affiliations:** 1 Department of Computer Science and Engineering, Indian Institute of Technology, Roorkee, 247667 India; 2 Bernoulli Institute of Mathematics, Computer Science and Artificial Intelligence, University of Groningen, 9700 AK Groningen, the Netherlands; 3 Department of Cognitive Psychology, Perception and Method Research, Institute of Psychology, University of Bern, 3012 Bern, Switzerland

**Keywords:** Neuroscience, Psychology, Computer science, Computational science

## Abstract

Lapses in attention can have serious consequences in situations such as driving a car, hence there is considerable interest in tracking it using neural measures. However, as most of these studies have been done in highly controlled and artificial laboratory settings, we want to explore whether it is also possible to determine attention and distraction using electroencephalogram (EEG) data collected in a natural setting using machine/deep learning. 24 participants volunteered for the study. Data were collected from pairs of participants simultaneously while they engaged in Tibetan Monastic debate, a practice that is interesting because it is a real-life situation that generates substantial variability in attention states. We found that attention was on average associated with increased left frontal alpha, increased left parietal theta, and decreased central delta compared to distraction. In an attempt to predict attention and distraction, we found that a Long Short Term Memory model classified attention and distraction with maximum accuracy of 95.86% and 95.4% corresponding to delta and theta waves respectively. This study demonstrates that EEG data collected in a real-life setting can be used to predict attention states in participants with good accuracy, opening doors for developing Brain-Computer Interfaces that track attention in real-time using data extracted in daily life settings, rendering them much more usable.

## Introduction

Attention plays a vital role in our day to day lives. It is essential for something as trivial as getting someone’s name right in a noisy place to something as crucial as avoiding making mistakes in a dangerous factory environment. Attention fluctuates over time^1^, and a sudden decrease can have disastrous consequences, for example leading to traffic accidents^2^, medical mistakes, factory incidents, etc. Given the importance of attention in everyday life, it would be helpful to track attentional states in real time. Various (neuro)physiological tools have been used for this end, including electroencephalography (EEG).

Mulholland^3^ demonstrated that when a person was not paying attention, 9–14Hz alpha power in occipital electrodes was higher compared to when they were paying attention. Jin et al.^4^ conducted a study with 18 participants using a $128+6 (EOG)$ EEG device. The participants performed a sustained attention and visual search tasks and self-reports of attentional state were used for labeling. They reported an accuracy of approximately $60\%$ using SVM for classifying self- reported attentional state, predominantly on the basis of occipital alpha oscillations. Another study by Mohamed et al.^5^ conducted with 86 participants using a 14 channel (dry) EEG device reported the maximum accuracy of 70% with SVM, KNN and Gaussian Process for classifying attention into three classes (high, low and medium). The participants performed tests^6^ containing activities that assessed attention, memory, perception, and other cognitive states. Awais et al.^1^ performed a driving simulator and EEG (20 channels with dry electrodes) study with 22 participants to differentiate between attention and drowsy states. The study was conducted in three phases. Familiarization (no data collected) phase to familiarize the participants with the simulator. Training phase was next that familiarized the participants with the simulator for 10 min while wearing an EEG cap. Then was the monotonous Driving(MD) phase where the participants drove a car for 80 min at a speed of 80 km/h. Video recording during the MD phase was used to label instances of interest by identifying drowsiness-related events using from facial features, including eye blink duration, facial expressions, facial tone, eye blinking rate, and movements such as head-nodding and yawning. They also used self-rating on a Karolinska Sleepiness Scale (KSS)^7^ at the start and end of the MD phase. They found that P3, P4, P7, P8, C3, Cz, O1 and O2 electrodes showed significant differences in signal amplitude between self-reported attentive and self-reported drowsy states. They also reported that EEG in every 10-min window showed differences in alpha, delta and theta frequency and SVM gave an accuracy of 80.60% in classifying alert and drowsy states.

Supplementary Table 1 provides a more detailed overview of these studies, which think form a representative sample of the broad literature. These findings have been replicated many times^4,5,8^ but most of these studies involve recording the EEG signals in a lab, comparing conditions during which participants are distracted with external stimuli to conditions in which they are not. The common findings from these studies include:

1. Delta (0–4Hz), theta (4–8Hz) and alpha (8–13Hz) waves are most related to attention amongst all the brain waves. Various studies report increased mid-frontal theta, decreased central and parietal delta and decreased frontal and parietal alpha power with attention^1,4,9–12^.
2. Non-periodic EEG activity in the parietal, occipital and fronto-central regions of the brain being associated with attention^1,9^.
3. EEG signals corresponding to attention and distraction can be classified using machine learning with accuracies of up to 89%^10,13^.

**Figure 1:**
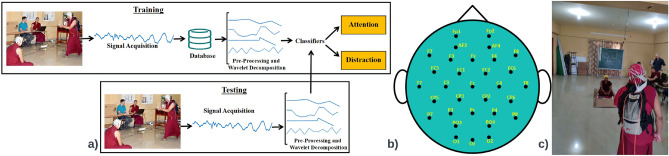
**(a)** Framework of the recognition system used for classifying attention and distraction states. **(b)** Electrode positions as per the 10–20 International system belonging to Biosemi 32 channel EEG device used for data collection. **(c)** Experiment setup. EEG being recorded from both the participants simultaneously during a monastic debate with raters of attentional state in the background.

It is important to note that besides most of these studies use the EEG data collected in a laboratory setup while participants are given computerized tasks with carefully-controlled stimuli, constraints are also placed on the movement (should be avoided), speech (should also be avoided), etc of the participants. This means that the inferences obtained may not generalize to the real-life scenarios in which we want to track attention for preventing errors or optimizing performance. Another issue is that most of these studies interrupt the task to obtain self assessment reports from the participants to derive the instances of attention (on-task) and distraction (off-task for example, mind wandering)^4,9,14^. This not only disrupts the attention of the participant during the task but these self reports can be biased and/or unreliable.

As an exception to this, Ko et al.^15^ conducted a semester-long EEG study with 18 participants in a classroom using a 32-channel gel-based EEG device. The data were collected while the participants had to detect special visual targets that occurred during regular university lectures as soon as possible. The authors reported that longer delays in reporting the visual targets were preceded by an increase in activity in the delta and theta bands and a decrease in activity in beta band over the occipital and temporal regions. Hence, while some studies^4^ are based on reliable data and a high number of participants, they are conducted in a strict laboratory setting and/or require the participants themselves to indicate their mental state at each moment , others are done in more naturalistic settings but are relying on lower number of participants and/or low-quality EEG signals^13,14^.

In this study, we explore if attention can be tracked in real-time in a complex real-life social situation using the EEG data collected without imposing any constraints on the behaviour of the participants. We also explore if attentional states can be distinguished if the labels are based not on the judgements provided by the participant themselves, but instead by a second-person observer. This second-observer method is much less intrusive and can even be done by post-hoc analysis of videos recorded during the task. Our aim was to collect EEG in a naturalistic environment with high-quality EEG equipment. Monastic debate practised by Tibetan monks in India is one relatively naturalistic context in which substantial variation in focus and distraction occurs. It is a contemplative debating practice that is different from the Western debate not only in terms of its physical setting (Fig. 1c) but also in its essence^16^. It is not aimed towards convincing the opponent of a standpoint but rather to find out inconsistencies in their reasoning. Monastic debates are accompanied by periods that are relatively boring since one goes through lines of reasoning systematically, as well as periods that are quite exciting when debaters tease each other or when they have almost demonstrated an inconsistency. We previously showed that monastic debate practice appears to enhance one’s neural signature of attentional focus, namely frontal midline theta power^16^, and therefore it is a good testing ground. Involving Tibetan monks as participants also helps us see if the findings can be generalised to a much different population than is usually studied (i.e., participants from Western industrialised countries)^17^. Monastic debate is explained in more detail in^16^.This study also tries to determine the differences (if those exist) in attention owing to experience of individuals in performing a task and if they can be captured by EEG data collected in a real-life scenario.

**Table 1:** Number of attention and distraction instances per debate in the dataset. The ’No.’ column represents the debate ID, ’Attention’ represents the number of attention instances and ’Distracted’ represents the number of distraction instances present in the particular debate. ’–’ denotes that no said instances were found where the majority of annotators agreed. If a debate ID is missing from ’No.’, it indicates that there were no labeled instances of attention or distraction in that debate.

No.	1	2	3	4	5	6	7	8	11	12	13	14	15	16	17	18	19	20	22	23
Attention	–	3	2	10	–	4	3	1	12	7	–	1	–	–	–	–	–	3	–	–
Distraction	10	3	4	3	11	–	7	1	–	1	9	13	10	15	12	6	12	1	12	12

No.	24	25	26	27	28	29	30	33	34	35	36	37	38	39	40	41	42	44	45	46
Attention	–	–	33	10	18	–	8	26	15	–	–	2	13	–	–	–	13	–	–	–
Distraction	11	5	1	2	5	5	–	–	6	12	4	9	4	11	7	16	-	21	36	5

## Methods

This section entails the description of the dataset, pre-processing, and other techniques used. The overall framework of the study can be seen in Fig. 1a.

### Participants

Given the limitations regarding budget and resources (specifically, the researchers had only limited time they could be present in the monastery in person for testing, and monks only had limited time available to participate in studies), data (which included not only EEG but also behavioral tasks and surveys) was collected from 24 participants. However, various other studies^12,18,19^ have also used data from 24 participants for their study and have given robust results.

In this study we focus mainly on the EEG data. 24 male Tibetan monks from Sera Jey monastic university in Bylakuppe, India, were recruited as participants in this study. Though monastic debate is a common practice in Tibetan Buddhist monasteries, this monastery was chosen because it has a large population of monks that are highly skilled in this form of debate^16^. Participants were encouraged to do two rounds of debates, but some pairs had to leave the session after a single debate due to other engagements. The participants took part in a total of 46 debates and aged between 20 and 30 years old.

### Ethics approval and consents

All procedures performed in studies involving human participants were in accordance with the ethical standards of the institutional and/or national research committee and with the 1964 Helsinki declaration and its later amendments or comparable ethical standards. The study protocol was approved by the CETO (Research Ethics Review Committee of the Faculty of Arts of the University of Groningen), protocol number 70890721. Oral informed consent was obtained from all individual participants included in the study. We chose to not use written informed consent because this would be very unfamiliar and anxiety provoking in this culture that is mainly oral. Permission for verbal consent was granted by CETO as well. It was emphasized that the participants could leave the study at any time if they desired to do so, without any repercussions. Informed consent was also taken for publication of identifying information/images in an online open-access publication.

### EEG recordings

The EEG signals were recorded using a Biosemi 32 channel EEG system and the electrodes according to the 10–20 International system are shown in Fig. 1b. Individual channels were adjusted until impedances were below 25 k$\Omega$^20^ and CMS-DRL (common mode/driven-right leg—standard for a Biosemi EEG device) was used as the reference. The sampling rate was 512Hz. EEG caps were mounted on the heads of both the participants and signals were recorded during the entire duration of the debate from both of them simultaneously. This led to a large dataset with 92 (46*2) EEG recordings.

#### Manipulation of difficulty

To examine how topic difficulty affected the neural correlates of debate, and the amount of attention and distraction, debates were conducted on easy and more difficult topics^16^ from their curriculum. There are a total of 46 debates (23 easy and 23 hard). Participants were assigned a specific debate topic and asked to debate for 10 min (easy debates) or 15 min (hard debates). These duration were chosen because they accord well with the natural duration of debates. However, because of the limited amount of data, the difficulty manipulation is not further examined in this study.

#### Variability in experience

Debaters with varying experience were involved in the study to examine the effects of debate training on cognitive and affective function. Monks were invited by class^12^. The class recruitment ensured that monks that have learnt how to debate, and have learnt the text we asked them to debate about. 10 more experienced participants with the monastic training of about 14–25 years and 14 less experienced participants with monastic training of 3–10 years participated in the study. Debaters were always paired with another debater who had the same level of experience because otherwise debates tend to not flow well.

### Video recordings

Video recordings (with audio) of all the debates were done to aid in annotating the instances of interest. Hence, it was crucial for the timing in videos and EEG signals to be synchronised. EEG and video recordings were started together with a countdown to 3 for 32 debates. However, an added measure for synchronization was employed for the remaining 14 debates. The EEG and video recordings were started on countdown once when the participants were ready for debate but at the start of the debate a participant from the debating pair was asked to slowly blink 5 times. The blinks which are easily detected in the raw EEG signals provided the offset in time between the EEG and videos recordings to millisecond precision. Example video recordings have been uploaded online^21,22^.

**Figure 2:**
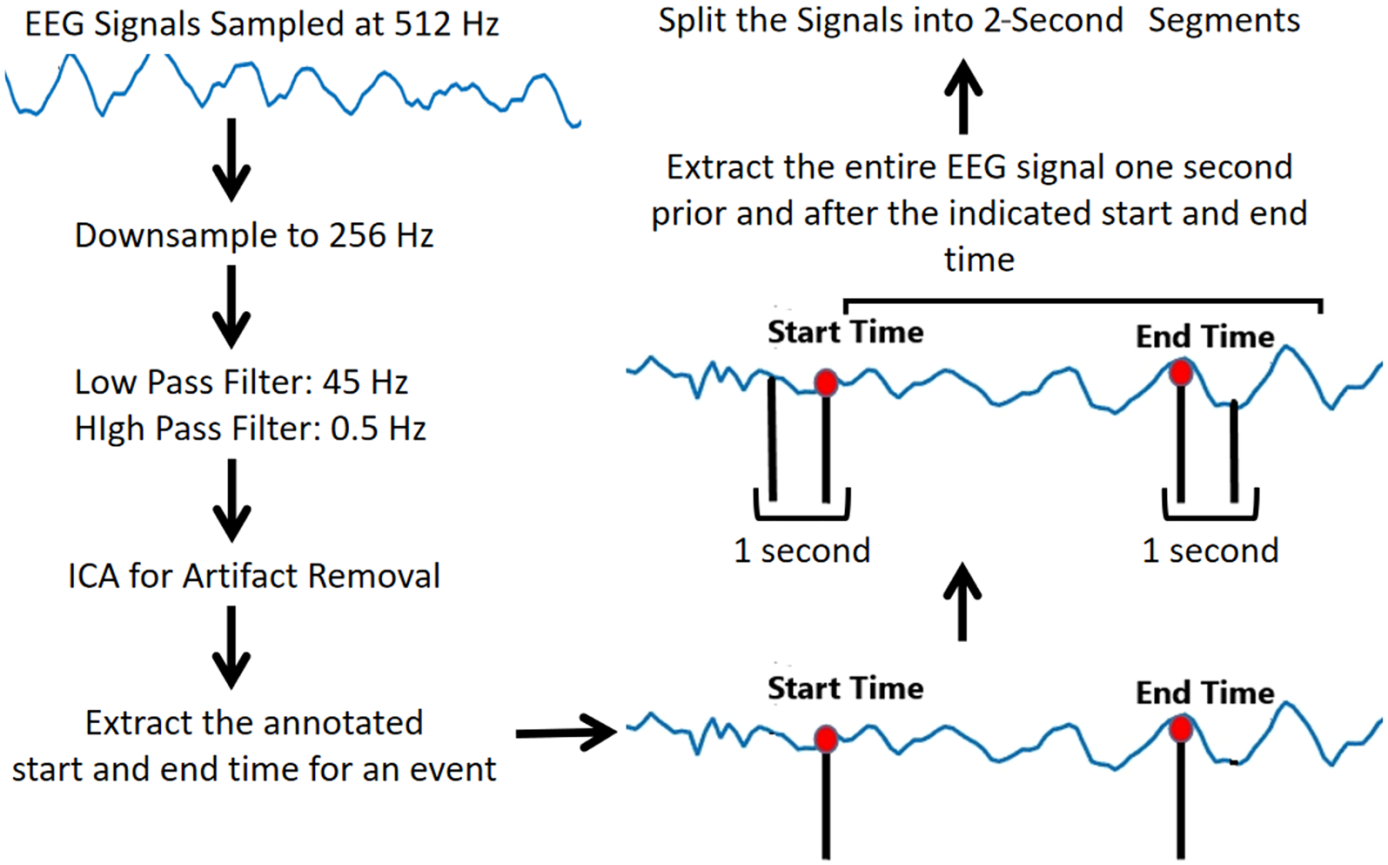
Steps followed for pre-processing the collected EEG data.

### Annotation of the data

To obtain ratings of attention and distraction, three senior monks, fluent in Tibetan and well versed in monastic debate observed the recorded videos. They used Behavioural Observation Research Interactive Software (BORIS) event logging software^23^ to indicate when the debaters exhibited signs of either intense attention (staying relevant to the task) or serious distraction based on their utterances and their body language, like eye gaze (whether they are averted from the debater), facial expressions (like confusion, if they are consistent with the ongoing dialogue), and content of utterances (whether the participant is replying to the question asked, they asked to repeat the question because they did not listen) to determine if the participant’s response was not at all linked to the posed question, etc for annotation. These cues are similar to what we use in a normal conversation to see if a person is listening attentively or not, but narrowed down to the specific context of monastic debate. BORIS allows the rater to mark the start and end of the time interval corresponding to a certain category with a millisecond precision. These senior monks were all expert debaters with at least 15 years of training and were subsequently extensively briefed the meaning of the categories they had to mark. There were differences in rater annotations, with in particular large differences in the level of detail of labelling: some labelled minute changes, others more globally. To ameliorate these effects, we went with majority rating. We only labeled instances as ‘attention’ when at least half of the raters considered them ‘attention’, and ‘distraction’ when at least half of the raters considered them ‘distracted.’ In case a majority was not established we did not consider that instance during the analysis. Since the ratings involve the specification of time intervals, it is not possible to compute inter-rater reliability by means of the usual methods such as kappa. More details on annotation are presented in^12^ and Supplementary Methods.

**Figure 3:**
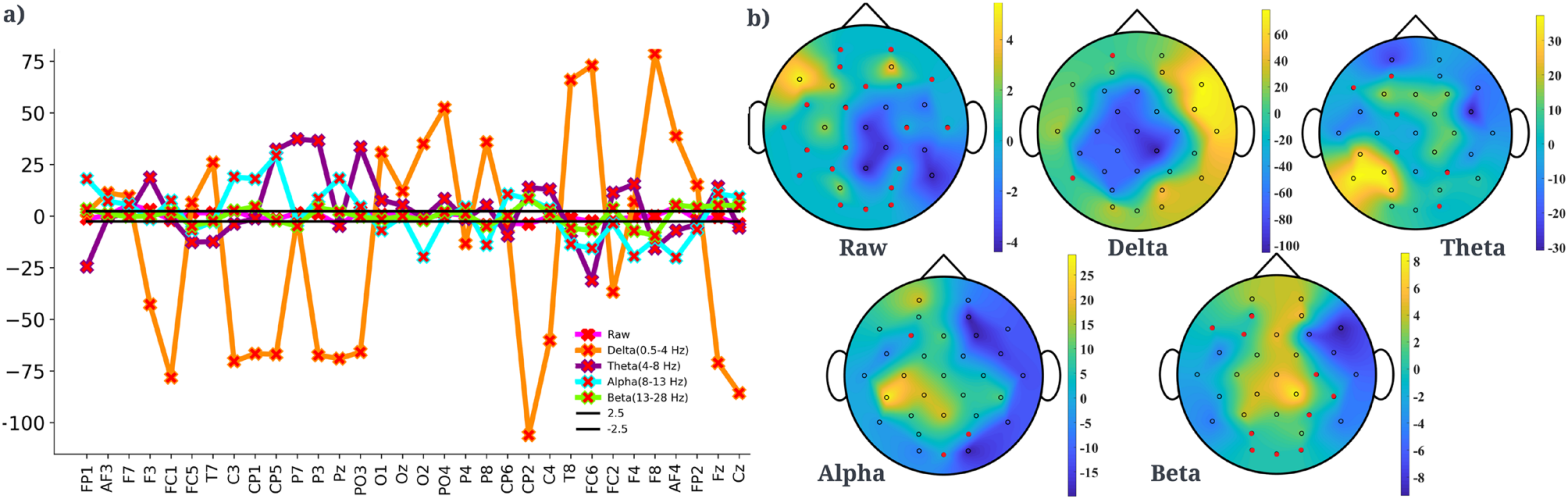
**(a)** t-value showing difference in mean amplitude (µV) between attention and distraction states in raw, delta, theta, alpha and beta brain waves data. Lines corresponding to 2.5 and − 2.5 are also shown for comprehension. **(b)** Topography plot of channels that show t-values of channels that had significant p-values of difference between attention and distraction in raw, delta, theta, alpha and beta brain waves after multiple correction using FDR (p-value threshold 0.0385). Channels with p-values above the threshold i.e. non-significant channels have been marked in red and corresponding t-values are plotted as zero for clarity.

### Pre-processing

Figure 2 shows the pre-processing steps. The EEG signals were downsampled to 256 Hz and a 0.5–45 Hz bandpass filter was applied to remove high-frequency muscle activity^24^. Following this, ICA was done by visual inspection of the EEG signals and the topographic maps to remove artifacts such as eye blinks, saccades, muscle activities etc. (similar to^12^). We also re-referenced the data to average reference which is preferred as it is a very stable referencing method^25^. To extract EEG segments corresponding to our events of interest, we extracted 2-s segments from EEG signals with 1 s prior to time indicated by the senior monks as reflecting focus and distraction. The reason for this timing is that it can account for the response time of the rater, which is on the order of 1 s. There are 142 and 46 segments of 2-s each corresponding to distraction and attention labels respectively in the easy debates and 160 and 138 segments of 2 s each corresponding to distraction and attention respectively in the hard debates. Thus, there are a total of 302 and 184 instances of distraction and attention, respectively.

Table 1 shows instances of attention and distraction per debate. These are the instances that the majority of annotators agreed upon and have been included in the study. On an average, there are 10 and 9 (rounded off) instances of attention and distraction per debate respectively. The range (maximum value − minimum value) of attention and distraction instances per debate is 32 and 35 respectively. Such substantial variation is expected, since some debates (conversations) are very engaging, while others are not.

### Wavelet transform

The cognitive states we are considering are not strictly time-locked to a particular moment and therefore it makes most sense to focus on brain oscillations that are not so strongly time-locked. Moreover, brain oscillations have been linked most strongly to attention and distraction, especially those in the alpha and theta bands^4^. Various studies have found wavelet transform as a more efficient method for spectral analysis.^26,27^ Daubechies (Db)-8 wavelet transform is used here^28^. A wavelet function at time *t* is shown in Eqs. (1) and (2), where $a= 0,1,\ldots ,A'-1$, $t= 0,1,\ldots , T-1$, $A' = log_2(t)$, $b= 0,1,\ldots ,2^a-1$, T is the length of the signal, $a_{0}$ and $b_{0}$ values are set to 2 and 1, respectively.

1 $$\begin{aligned}{} & {} \int _{- \infty }^{\infty } \psi (t) \, dt = 0 \end{aligned}$$

2 $$\begin{aligned}{} & {} \psi _{a,b}(t) = a_0^{{-a/2}} \psi ( a_0^at-bb_0) \end{aligned}$$

**Figure 4:**
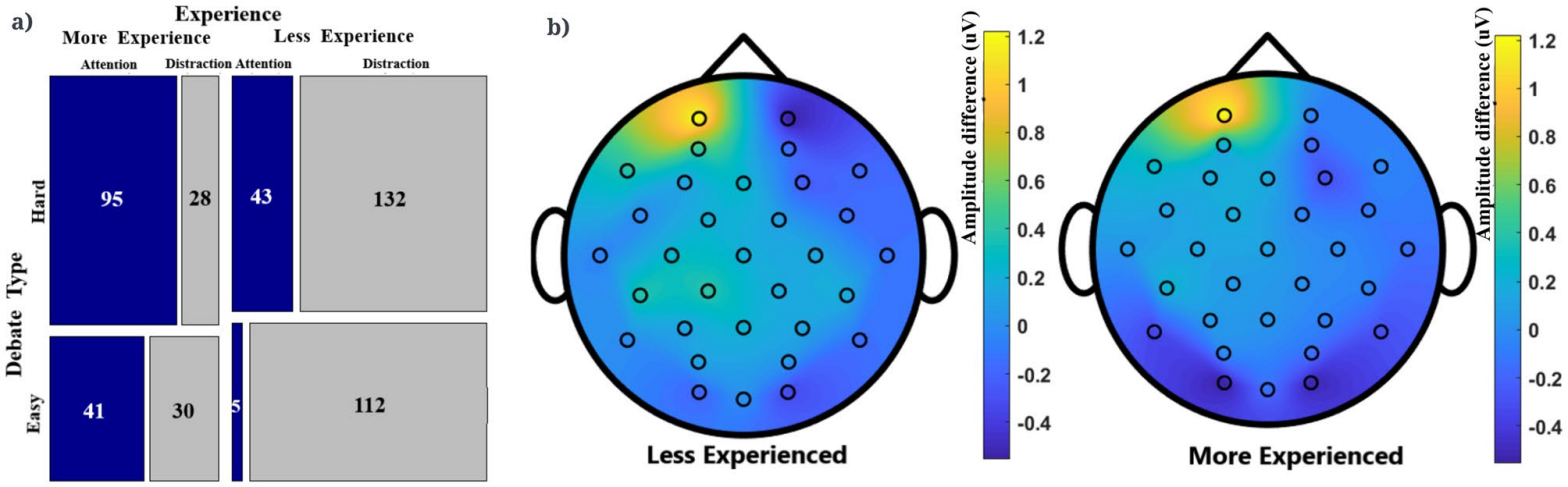
**(a)** Effect of debate experience on the frequency of occurrence of instances of attention and distraction. **(b)** Topography maps comparing alpha activity corresponding to the difference (µV) between attention and distraction states for less and more experienced participants.

The scaling and wavelet functions required for evaluating the Approximation (A) and Detail (D) coefficients is given in Eqs. (3) and (4), respectively.

3 $$\begin{aligned}{} & {} \phi _{p,q} (t)=2^{p/2} h(2^pt-q) \end{aligned}$$

4 $$\begin{aligned}{} & {} \omega _{p,q} (t)=2^{p/2} g(2^pt-q) \end{aligned}$$

The $A_i$ and $D_i$ coefficients at the $i^{th}$ level are evaluated using Eqs. (5) and (6), respectively.

5 $$\begin{aligned}{} & {} A_i=\frac{1}{ \sqrt{T}} \sum _t x(t) \phi _{p,q} (t) \end{aligned}$$

6 $$\begin{aligned}{} & {} D_i=\frac{1}{ \sqrt{T}} \sum _t x(t) \omega _{p,q} (t) \end{aligned}$$

The Db-8 decomposition renders five wavelet coefficients corresponding to delta (0.5–4 Hz)^29^, theta (4–8 Hz)^30,31^, alpha (8–13 Hz)^32^, beta (13–28 Hz)^31^, and gamma (28-45 Hz)^31^ brain waves. Since various studies have reported gamma waves to be associated with motor movement, and as no restrictions were placed on the movement of the participants in this study, those were excluded from further analysis^33,34^.

## Results

In this section we first examine what electrodes and frequency bands show differences between attention and distraction on average. Then, we examine whether it is possible to make use of these data to predict attention and distraction in real-time on a single-trial level using machine/deep learning.

### Statistical differences between attention and distraction

In order to estimate the sufficient number of sample size for this study we conducted a power analysis. The reported values in^4^ result in an effect size of 0.99, suggesting a minimum sample size of 11 at a power of 0.842. As the sample size for our study is 24 this results in a power of 0.91 which is sufficient to detect the difference between the two groups—attention and distraction.

An independent t-test (t $\ge$ 4, threshold of 4 was chosen to reduce false positives) on the EEG data showed that in addition to alpha, significant differences between attention and distraction states exist in the raw, delta, theta, and beta frequency bands. Figure 3a shows the t-values corresponding to the raw data and brain waves. However, an issue with EEG studies is that there are many electrodes and when statistical tests are done on each individual electrodes for each brain wave, that leads to an inflation of the false positive rate. To correct for this, we used the False Discovery Rate (FDR)^35^. A false discovery threshold level of 0.05 was used for the analysis which corresponds to the p-value threshold of 0.0385. Delta and alpha brain waves yielded the maximum number of channels that are significant in distinguishing attention from distraction. This analysis also shows that delta, alpha and theta brain waves have a more widespread area of channels that show a significant difference in activity between attention and distraction states. Delta and alpha brain waves have significant channels in the frontal and parietal areas of the brain and theta wave in right frontal, temporal and left parietal areas. The associated topographical maps with t-values of channels with significant p-values (after FDR) are shown in Fig. 3b.

**Figure 5:**
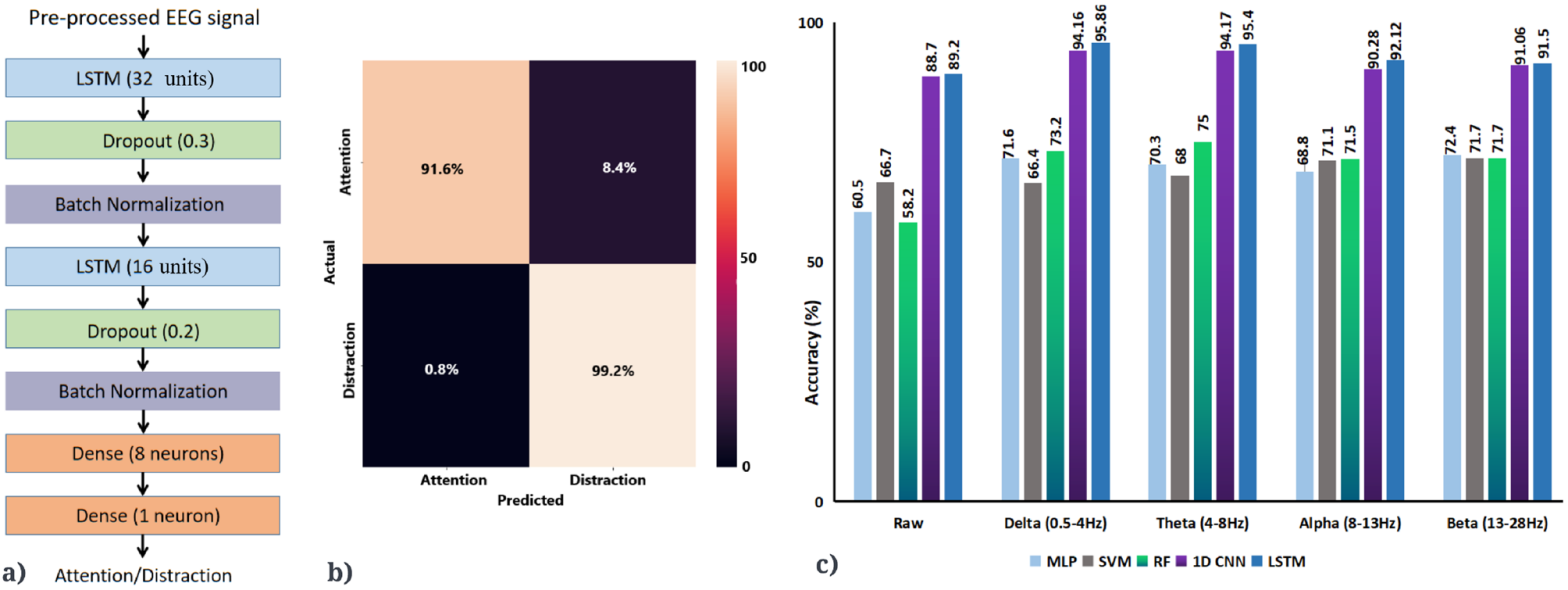
**(a)** LSTM architecture that predicted the attentional states with best accuracy. **(b)** Confusion matrix corresponding to test data in theta band for the dataset using LSTM which gave the maximum accuracy out of the various classifiers that were tested. **(c)** Comparison of classification accuracy on the basis of different data aspects for the test dataset. Delta (0.5–4 Hz) and theta (4–8 Hz) activity were most predictive of attentional state.

### Differences in the states of attention and distraction associated with the differences in experience in the participants

It is of interest to know whether practice at a task (here: debate training) reduces distractability. To address this question we compare more and less experienced participants. The 23 easy debates in this dataset consisted of 11 debates amongst more experienced and 9 debates from less experienced participants. The 23 hard debates comprised of 7 and 14 debates between more and less experienced participants, respectively. It was found that both the hard and easy debates have more instances of distraction than attention. However, as one might expect, less experienced participants had more instances of distraction and fewer instances of attention than the experienced participants. Figure 4a shows the count of these instances varying across the hard and easy debates amongst participants with different experience. A chi-square test ($\chi ^2$ (1) = 140.41, p < 2.2e−16) on these proportions (not taking the debate type into account) suggests that the proportions of attention vs distraction differ between beginners and more experienced participants.

Figure 4b shows that for the alpha band, more experienced participants had higher activation in the occipital region when they were distracted as opposed to higher activation in temporal, occipital and frontal regions for the less experienced participants. Both the categories showed higher activation in the left pre-frontal region when they were rated to be attentive.

### Machine learning to predict attention and distraction using EEG signals collected in a real life setting

Having established that there exist average differences between attention and distraction in the dataset, which predominantly manifest in right frontal channels, particularly in the alpha and theta bands, we then asked whether the mental state could be classified on a single-trial level in real-time. In order to classify the EEG signals as attention or distracted we experimented with various classifiers, Support Vector Machine (SVM), MultiLayer Perceptron (MLP)^36^, Random Forest (RF)^37^, 1D-Convolution Neural Network (CNN)^38^ and Long Short Term Memory (LSTM)^39^. However, there is an imbalance in the dataset which has 340,480 samples for distraction as opposed to 164,864 samples for attention. This imbalance can easily lead to bias and lack of generalization in the classifiers. To avoid the bias, 200,000 instances of distraction were randomly sampled and used for classification.

For machine learning algorithms (MLP, SVM, RF) k-cross validation (k = 5,7, and 10) was used with k = 10 giving the maximum accuracy. Since hyper-parameter tuning is required for deep learning (CNN and LSTM), data were split into training, validation and test set in the ratio of 60:20:20 respectively^40,41^. We experimented with various models, trying different permutations of layers, regularizers, optimizers, batch size, window size and activation function. The models that performed the best are reported in the manuscript.

A 3-layer MLP with 16, 8 and 4 neurons in each layer, learning rate of 0.001, batch size 32, and Adam optimiser^42^(alpha = 0.001, beta1 = 0.9, and beta2 = 0.999) gave the highest accuracy of $72.4\%$ in the beta wave. SVM with kernel as radial basis function gave the highest accuracy of about $71\%$ in the alpha and beta wave. The ensembling technique of random forest of 50 decision trees each with maximum depth of 16 and Gini impurity criterion gave the maximum accuracy of 75% in the theta wave. 1D-CNN layer with 512 filters and 5 kernel size, followed by batch normalization^43^, dropout^44^(0.4) and a fully-connected layer with sigmoid activation gave the maximum accuracy of $94.17\%$ in the theta wave. After experimentation the LSTM architecture in Fig. 5a was found to give the best accuracy of accuracy of 95.86% and 95.4% for delta and theta waves. The learning rate, batch size, optimizer, sequence size for both reported models of 1D-CNN and LSTM were 0.001, 32, Adam^42^ (alpha = 0.001, beta1 = 0.9, and beta2 = 0.999) and 32 respectively. Figure 5c summarises the results obtained from various classifiers for each brain wave. LSTM predicts the states of distraction with a high accuracy of 99.2% but classifies approximately 8.4% of attention labels as distraction as shown in the confusion matrix corresponding to the theta wave in Fig. 5b. Refer to the Supplementary Methods for more information on classifiers.

**Figure 6:**
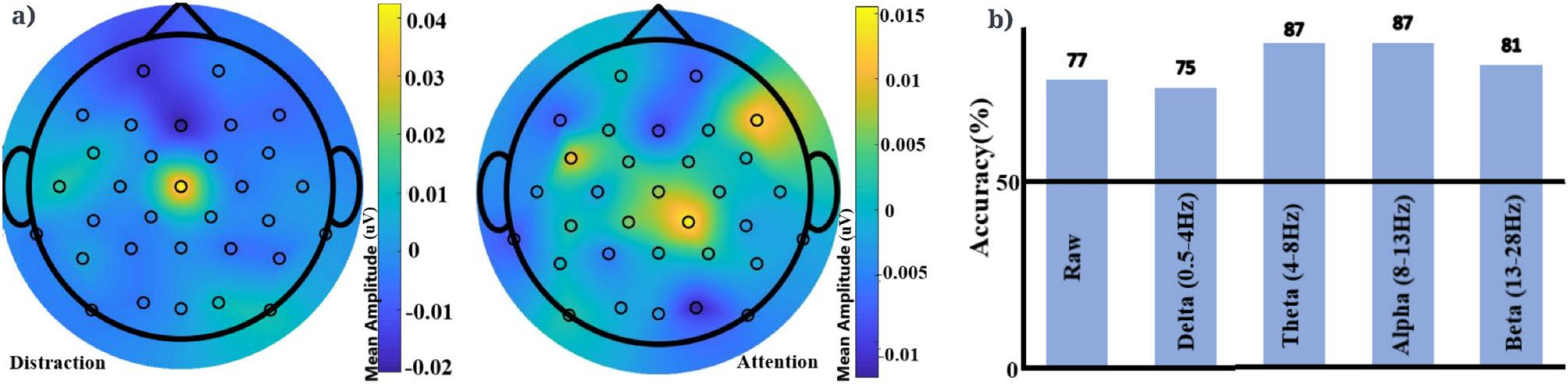
**a)** Topography maps of mean amplitudes (µV) corresponding to distraction state (left) and attention state (right) in alpha wave for dataset ’B’. **b)** Classification accuracy on the basis of different data aspects for the dataset ’B’ using the random forest classifier.

### Towards more generalization

Precise annotation of a dataset plays a crucial role in classification using supervised learning. However, it is not always possible or easy to have these precise annotations when dealing with data acquired in a real-life scenario. In order to check if attention and distraction can be detected in a dataset that is not precisely annotated, we worked with a similar dataset (referred to as dataset ’B’ to avoid confusion) mentioned in^16^. It is a larger dataset with EEG data and video recordings from 55 Monastic debates. However, since that dataset was focused on finding differences between agreement and disagreement rather than focus and distraction, which were encoded incidentally made it less specific and accurate than dataset ’A’ reported above.

Moreover, it incorporated quite an inaccurate method to match the timing of EEG signals to the videos which can lead to some discrepancies in annotation. Dataset ’B’ showed differences in mean amplitudes between the two mental states but the topography maps did not show any noteworthy differences between various brain areas in raw or brain waves as shown in Fig. 6a. Nevertheless a RF classifier of 20 trees with maximum depth of 20 still gave a maximum accuracy of 87% in the alpha and theta waves as shown in Fig. 6b. As this dataset is noisy, random forest was chosen for classification as many studies have reported it to be robust to noisy data.^45,46^ It is difficult to know whether the EEG signals corresponding to attention and distraction are sufficiently distinctive to be captured by machine learning even when the annotations are not as precise on the time axis or whether instead the classifier is picking up on noise in the EEG signals.

To address that question, we attempted to generalize between the classifiers of datasets ’A’ and ’B’. Specifically, we trained the random forest classifier on dataset ’B’ and tested it on the dataset ’A’. The classifier had 100 decision trees and each tree had a maximum depth of 40 but an accuracy of only 53% was obtained on the test set. Reversing the role of the datasets as train and test sets yielded a below-chance accuracy for the two mental states. It suggests that although the attention and distraction instances in the debaters in the individual datasets could be predicted, those predictions cannot be used to generalize these two cognitive states at least not for the datasets currently available. The transfer might be possible if a high-quality dataset were available.

## Discussion

Monastic debate was chosen as the task in our study as it is not only a less studied form of debate but also is a good testing ground for attention and distraction. We deliberately did not interrupt the flow of any debate or impose any restrains on the participants in terms of movement, speech, etc. so that the findings from the study can potentially be generalised to other real life scenarios like classrooms, tracking attention to study medical conditions such as Attention Deficit Hyperactivity Disorder (ADHD), etc.

Previous studies pertaining to understanding attention using EEG, found that theta and alpha bands are the most informative. Hence, we limited ourselves to these features. An important reason for doing so is the risk of overfitting in such a small dataset. It has been reported that adding too many features will inflate the false positives and lead to multiple comparison issues^47,48^. Additionally working with band waves is more interpretable by neuroscientists than features like mean, root mean square, etc., and thereby helps to understand the working of the brain.

We found that when participants were in an attentive state, their left frontal alpha had a higher amplitude compared to when they were distracted. Surprisingly, these findings are at odds with laboratory studies that associate attention mostly with decreased posterior alpha amplitude. A possible explanation for these findings could be that the distraction states that are observable overtly in a real-life situation are different from the self-reported distraction states in a controlled laboratory situation. However, in addition, attention was associated with decrease in central delta and increased left frontal theta wave amplitudes which has been reported by other studies as well^1,35^. This indicates the possibility of delta and theta brain waves being more reliable indicators of attention in laboratory and real-life settings, however, more studies need to be done to corroborate these results.

An important innovation in our study was to determine attentional state on the basis of ratings of videos, using a detailed rating scheme as opposed to self-reports which disrupt the attention of the participant from the task itself. The second-observer method used in this study has the benefit of not disturbing the debaters during the task. However, since attention and distraction are mental states known best to the person himself, only strong instances of attention and distraction could be annotated. Although this annotation method is more in line with how people figure out if a person is attentive or not in their day-to-day life, but, as one is more sure of someone being distracted than attentive, this resulted in a small dataset and a substantial imbalance in the dataset with relatively more instances of distraction reported than attention. To address this, one needs to ensure that the task is long enough that enough relevant samples have been collected. In our study, we found that 10 min of debates garnered enough samples to work with machine/deep learning.

It was also examined whether experience of an individual affected these mental states in terms of number of occurrences or their neural correlates. Not surprisingly, less experienced participants showed more instances of distraction both in easy and hard debates than more experienced monks consistent with the idea that debate helps to train attention^8^.

In order to identify the differences between attention and distraction, statistical analysis and machine/deep learning classification was carried out. The classification results were corroborated by statistical analysis, which also showed that delta, theta and alpha oscillations have most statistically significant electrodes that differentiate attention from distraction. It was found that LSTM classifier was best at predicting the instances of attention and distraction in the EEG data and obtained an accuracy of 95.86% and 95.4% in the delta and theta brain waves respectively. LSTMs outperforming other classifiers in predicting the subtle attention states using EEG is reassuring, given that LSTMs have been proven to work very well for predicting time-series data. This reinforces the idea that LSTMs are suitable for similar problems. This can prove to be a stepping stone in developing BCIs^49,50^ that predict attention of users in real-time and in real-life scenarios like classrooms where the parameter of how attentive a student has been in class, is generally found by the grades they get. This system may be used in predicting if students are really attentive in the classrooms and hence can help in finding students who might need extra help. Such a BCI can potentially be used in conjunction with other sensors like EKG, etc and may be incorporated in vehicles so that vehicle can automatically stop (or go to self driving mode, etc) if the driver is distracted for a long duration. It can be used for factory workers where distraction leads to loss of lives or money so that such instances are detected and tended to before any loss is incurred. Nevertheless, before such exciting applications are possible, our findings should be replicated in different scenarios and populations.

## Conclusion

With this study we tried to cover the gaps in previous studies conducted for differentiating between attention and distraction by working on EEG data collected from a real-life scenario of monastic debate with a high-quality 32-channel EEG recording system. It was found that on average the data showed significant differences between the states of attention and distraction suggesting that EEG data collected in real-life scenarios can help predict attention and distraction. Attention was on average associated with higher left frontal alpha and left parietal theta power. Attention was also associated with a decrease power in central electrodes in delta wave. Classification was performed with support vector machine, multilayer peceptron, random forest, 1-D CNN and LSTM. We found that the highest classification accuracy (approximately 95%) was observed with LSTM on the basis of delta and theta activity.

## Supplementary Information

Supplementary Information.

## Data Availability

Pre-processed EEG data can be downloaded from: https://unishare.nl/index.php/s/1UYBgoG7tF2xfqG Scripts are available at: https://github.com/kaushik-pallavi/scripts_monks.
