# Supplementary material for: Decoding the cognitive states of attention and distraction in a real-life setting using EEG
Source: Sci Rep. 2022 Nov 30;12:20649. doi: 10.1038/s41598-022-24417-w (PMC9712397; doi:10.1038/s41598-022-24417-w)
Supplement: Supplementary file 1 — Supplementary Information. [file 41598_2022_24417_MOESM1_ESM.pdf]

Supplementary Material: Decoding the Cognitive States of Attention and Distraction in a Real-Life Setting Using EEG

Pallavi Kaushik[1,2,*], Amir Moye[3], Marieke van Vugt[2], and Partha Pratim Roy[1]

[1]Department of Computer Science and Engineering, Indian Institute of Technology, Roorkee, 247667, India
[2]Bernoulli Institute of Mathematics, Computer Science and Artificial Intelligence, University of Groningen, 9700 AK, the Netherlands.
[3]Department of Cognitive Psychology, Perception and Method Research, Institute of Psychology, University of Bern, 3012 Bern, Switzerland
[*]pkaushik@cs.iitr.ac.in/p.kaushik@rug.nl

Supplementary Table 1: Prior work on tracking attention using machine learning classifiers (P = Number of participants, C = EEG Channels and E = Electrode type)

**Ref.**	**P, C, E**	**Task description**	**Generation of labels**	**Findings**
Taillez et al. [1]	P:16 C:84 E:Wet	To focus on speech of one speaker in a two-speaker environment. Task was paused and listeners were asked to answer questions based on the content every 10 minutes (5 times)	The level of attentiveness	P9/ TP7/ T7 and TP10/ TP8/ T8 channels were most relevant for classification
Moon et al. [2]	P: 18 C: 14 E: Dry	Shown video clips to understand the engagement. Questions asked about various video segments to judge their level of attentiveness.	Correct answers were judged as "attentive"	1) Channels in left hemisphere of fronto-central, parietal, temporal, and occipital lobe found crucial. 2)Random forest provided accuracy of 52.96%
Liu et al. [3]	P:24 (students) C:1(FP1) E:Dry	1) To listen to English conversations before answering questions. 2) Distraction of two people while conversing. To answer questions and to report the content of the overheard conversation.	Tasks 1 and 2 were hypothesised corresponding to attentive and inattentive states respectively. Experiments were video recorded. Videos were watched to determine if the participant was attentive or not.	1)SVM provided the classification score of attentive and distracted states with 76.82%. 2) Delta brain wave found to be crucial for classification
Ghasemy et al. [4]	P: 10 (visually impaired students) C: 12 E: Dry	1) Task session- To listen to an audio lecture (about 7 minutes) on a topic attentively. 2) Mind wandering session- To focus on anything and relax while nothing was played.	1) After the lecture, participants were asked whether they were attentive; after the mind-wandering session participants were asked whether they succeeded in mind-	1) Alpha, delta and theta/low beta waves were indicative of attention. 2) O1, O2, P7, FC5, P8, F8 and F7 channels were related to attention. 3) LDA, SVM and k-NN were used for

			wandering.<br>2) Facial expressions and gestures were considered. Only data<br>in which subjective judgements<br>agreed with facial expressions and gestures were included.	classification. LDA provided best results with 89%
Abeer [5]	P: 5<br>C: 12<br>E: Dry	First ten minutes of each trial: Focused control of a simulated train. Remaining time of the trial: stop following the simulator and become unfocused with respect to changes on their computer	Attention: first ten minutes of a trial<br>Distraction: remaining time of the trial	Random forest achieved an accuracy of 96%
Çiğde-m İnan [6]	P:5<br>C: 11<br>E: dry	First ten minutes of each trial: Focused control of a simulated train. Next ten minutes: stop following the simulator and become unfocused with respect to changes on their computer. Last ten minutes: allowed to relax freely, close eyes and doze off, as desired	Attention: first ten minutes of a trial<br>Distraction: next ten minutes<br>Drowsiness: lat ten minutes of the trial	1) Brain activity in parietal and frontal lobes in delta, theta and alpha bands were associated with the changes in attention state.<br>2) SVM gave the maximum accuracy of 96.70%
Suhail et al. [7]	P:33<br>C: 128<br>E: wet	Focused reading task: read a printed scientific article related to the basics of brain functions for five minutes.<br>Resting: 5 minutes of no movement and relaxation	Attention: focused reading task<br>Resting: resting task	97.85% (Resting vs Focused Reading) using SVM

Supplementary Methods

Annotation of data

Attention and distraction have many interpretations. Since this is a fairly real-life situation, we use colloquial meanings of attention and distraction. In the case of monastic debate, we define distraction as attending to something that is not the main task (in this case the current debate). The distraction could be perceptually guided (i.e., an external event) such as the sound of a cow in the courtyard or self-generated (i.e., an internal event) such as a daydream. Furthermore, both zoning out (being distracted without awareness of drifting off) and tuning out (deliberately attending to task unrelated stimuli) would count as distraction. Attention would include attending to task relevant, self-generated information (e.g., thoughts about what to say next) as well as task-relevant, perceptually guided information (e.g., what the opponent is saying, or how his facial expression changes, etc.). In short, the essential difference between attention and distraction is that the former orients towards the task-relevant while the latter does not. Thus, for this study, the connotation of attention is similar to its common-sense definition that we use in our day-to-day lives.

The following table summarizes the cues used for annotation of the data.

Cues	Behavior
Eye gaze	Upward, directed away from the debater for a long time

Facial expressions	Confused, distracted
Content of utterance	whether the participant is replying to the question asked, whether they are consistent with the ongoing dialogue, asking for repetition of what the other debater said

Classifiers

Support Vector Machine (SVM)

It is a popular supervised learning algorithm that is used both for classification and regression. SVM plots the data points (x_1, x_2, ..., x_n, 'n' being the number of features) in an n-dimensional space and finds a hyperplane or a set of hyperplanes that separates the support vectors by maximum distance. For handling non-linear data, kernel functions are beneficial. They allow mapping non-linear data to a higher dimension where they are linearly separable, which can then be classified by SVM.

MultiLayer Perceptron (MLP)

This classifier comprises of an input layer consisting of 'n+1' neurons (data (x_1,x_2, ..., x_n) and a bias 'b'), 'i' hidden layer neurons and 'c' output layer neurons ('c' being the total classes in the dataset). Each neuron in a layer is a weighted sum of inputs from the previous layer followed by a non-linear activation function, eg for Suppl Fig. 1 ($h_i = tanh(w_1*x_1+w_2*x_2+...+w_n*x_n+b_1*w_0)$).
Output (Y) is determined at the last layer and error or loss is evaluated using the ground truth (Y') for each data point. Backpropagation algorithm [8] uses these errors to train MLP i.e. finetune the weights to optimize classification performance.

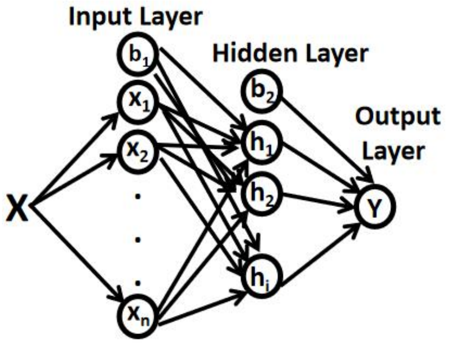

Suppl Figure 1. A multilayer perceptron

Random Forest (RF)

It is an ensemble technique that makes use of many decision trees for classification. Many decision trees are trained independently and the final decision of the RF is based on consensus. During the training phase, each decision tree's training set is created by random sampling. Addition of more decision trees decreases the variance in the model, however if the correlation between any decision trees increases, the error rate of the model also increases [9]. Thus, number of decision trees forming a forest is also a hyperparameter. Using many decision trees for classification, a RF classifier is good at dealing with noisy and missing data.

1D - Convolutional Neural Network (CNN)

CNNs are rooted in image processing as they were primarily used for image classification. They are known to capture the spatial features in data using a kernel that makes it robust at detecting distribution of colours, detect edges, etc. However they are no longer limited to handling images. 1d - CNNs, are suitable to work with time series data as their kernel moves along one dimension. CNNs typically consist of a convolutional layer (learns the features from the input), pooling layer (reduces the size of the feature maps while preserving important features), and fully connected layers (connecting the previous layer to the output neurons).

Long Short Term Memory (LSTM)

It is a recurrent neural network that tracks long-term dependencies in the input data leading it to predict the time-series data well. A basic LSTM unit consists of memory cells where each cell comprises of 3 gates, namely input, output and forget gate. The cell is responsible for handling the long term dependency while the three gates regulates the flow of values between the different layers of the LSTM network. The basic architecture of a single LSTM cell is as shown in Suppl Fig. 2, where x^t, c^t, h^t, c^{t-1} and h^{t-1} represent the previous input, current cell state, current output, previous cell state and the previous output, respectively. σ represents the sigmoid activation function, whereas '*' and '+' represent the mathematical operations of multiplication and addition, respectively. f^t, i^t and o^t represent the forget, input and output gate respectively.

The forget gate decides what previous information should be forgotten. Its output is formalised in Suppl equation (1).

$$f_t=\sigma(x_t*U_f+h_{t-1}*w_f) \qquad \text{Suppl (1)}$$

Where U_f and w_f denote the weight associated with the input and hidden state respectively.

The input gate is used to quantify the importance of the new information brought in by the input. It is represented by Suppl equation (2).

$$i_t=\sigma(x_t*U_i+h_{t-1}*w_i) \qquad \text{Suppl (2)}$$

U_i and w_i represent the weights associated with the input and the hidden state respectively.

The output gate is represented by Suppl equation (3).

$$o_t=\sigma(x_t*U_o+h_{t-1}*w_o) \qquad \text{Suppl (3)}$$

U_o and w_o represent the weights associated with the input and the hidden state respectively

The current hidden state h_t is given by Suppl equation (4)

$$h_t=\tanh(c_t)*o_t \qquad \text{Suppl (4)}$$

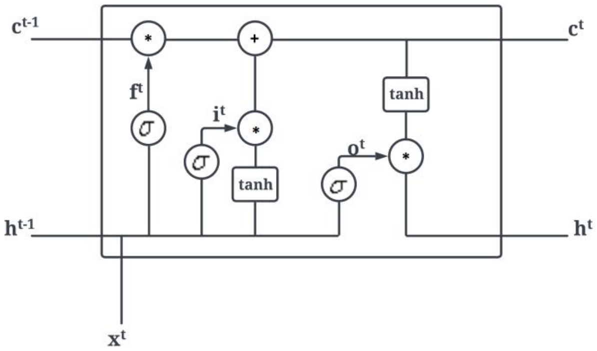

Suppl Figure 2. A LSTM cell in our network

A LSTM layer consists of 'n' units as shown in Figure 5a of the manuscript. A unit is a hyperparameter that determines the dimension of cell state and hidden state or more simply the number of parameters passed to the next LSTM cell. So the output dimension of a LSTM layer with 'n' units to the next LSTM layer will be (batch size, window size, 'n') while to a dense layer it will be (batch size, 'n').

Input to the classifiers

The dataset obtained after the ICA is termed as 'raw' in the manuscript. After ICA five brain waves were extracted. Each of the brain waves data were treated as a separate dataset for classifiers and hence the results are reported for each brain wave in Figure 5 of the manuscript.

For deep learning classifiers (LSTM and 1D-CNN) data with window size of 32 were prepared. This yielded blocks of data in the form of (block number * 32 (window size) * 32 (channels)). There was one label corresponding to each block hence it was made sure that the data under a label are put in one block which preserves temporal dependency in the data. So, 32 samples corresponding to attention were clubbed together as one block with label 'attention' while 32 samples corresponding to distraction were clubbed together as a different block with label 'distraction'. All the blocks were then

shuffled and randomly split into train, validation and test sets with the ratio of 60:20:20 and were fed to the model.

For machine learning classifiers, the data were shuffled and then 10-fold cross validation was performed.

More details can be found in the scripts uploaded on Github: https://github.com/kaushik-pallavi/scripts_monks

References
[1] de Taillez, T., Kollmeier, B. & Meyer, B. T. Machine learning for decoding listeners' attention from electroencephalography evoked by continuous speech. European Journal of Neuroscience 51, 1234–1241 (2020).
[2] Moon, J., Kwon, Y., Park, J. & Yoon, W. C. Detecting user attention to video segments using interval eeg features. Expert Systems with Application 115, 578–592 (2019).
[3] Liu, N.-H., Chiang, C.-Y. & Chu, H.-C. Recognizing the degree of human attention using EEG signals from mobile sensors. Sensors 13, 10273–10286 (2013).
[4] Ghasemy, H., Momtazpour, M. & Sardouie, S. H. Detection of sustained auditory attention in students with visual impairment. Iranian Conference on Electrical Engineering (ICEE), 1798–1801 (IEEE, 2019).
[5] Al-Nafjan, A., & Aldayel, M. Predict Students' Attention in Online Learning Using EEG Data. Sustainability, 14(11), 6553 (2022).
[6] Acı, Ç. İ., Kaya, M., & Mishchenko, Y. Distinguishing mental attention states of humans via an EEG-based passive BCI using machine learning methods. Expert Systems with Applications, 134, 153-166 (2019).
[7] Suhail, T. A., Indiradevi, K. P., Suhara, E. M., Poovathinal, S. A., & Ayyappan, A. Distinguishing cognitive states using electroencephalography local activation and functional connectivity patterns. Biomedical Signal Processing and Control, 77, 103742 (2022).
[8] Rumelhart, D. E., Hinton, G. E. & Williams, R. J. Learning representations by back-propagating errors. Nature 323, 533–536 (1986).
[9] Zhao, X. et al. Classification of sleep apnea based on eeg sub-band signal characteristics. Sci. Reports 11, 1–11 (2021).
